# Barriers to Breast, Cervical, and Colorectal Cancer Screenings Faced by Refugees Resettled in the United States: A Rapid Review

**DOI:** 10.1007/s10903-025-01690-1

**Published:** 2025-05-28

**Authors:** Jamie Nassur, Devesh Dajee, Amy Leader, Katherine DiSantis

**Affiliations:** 1 College of Population Health, Thomas Jefferson University, Philadelphia, USA; 2 Sidney Kimmel Medical College, Thomas Jefferson University, Philadelphia, USA

**Keywords:** Refugees, Cancer screening, Barriers, Trauma, Preventive health

## Abstract

Millions of refugees fleeing countries with political unrest and war have been resettled in the United States (U.S.). Although refugees resettled in the U.S. are granted access to health insurance, there are large disparities in the use of preventive health services like cancer screenings between refugees and the native-born population. The U.S. Preventative Services Task Force (USPSTF) recommends screening for breast, cervical, and colorectal cancers, which are highly preventable if detected early. Previous research has investigated barriers to screening for breast, cervical, and colorectal cancers that may underlie screening disparities. However, current reviews assessing these barriers do not distinguish between immigrant and refugee populations and primarily cover breast and cervical cancer screenings. This rapid review aimed to identify barriers to breast, cervical, and colorectal cancer screenings experienced by refugee-specific communities resettled in the U.S. Of the 945 articles retrieved from the database searches, 12 articles were selected for analysis. Data extracted from the selected studies included screening barriers, screening rates, and sample demographics. Six key barrier themes were identified: (1) language and health system navigation, (2) cost, transportation, and time, (3) health knowledge and exposure, (4) fear of cancer screening and diagnosis, (5) cultural and religious beliefs, and (6) refugee experience or status. We noted variations in the screening barriers experienced by different refugee ethnic groups and noted the impact of refugee experiences and trauma in shaping refugee perspectives on screening. The findings of this review suggest the need for interventions aimed at increasing screening rates to consider the nuances in perceived barriers by different refugee ethnic groups and the role of trauma associated with refugee experiences in shaping these barriers.

**Supplementary Information:**

The online version contains supplementary material available at 10.1007/s10903-025-01690-1.

## Introduction

Breast and colorectal cancers are among the most incident cancers in the United States (U.S.), representing nearly 40% of new cancer cases annually in females and 8% in males [1]. They account for about 23% of cancer deaths in females and 9% of cancer deaths in males [1]. Cervical cancer is less incident and has seen declines in cases partly due to the successful uptake of human papillomavirus (HPV) vaccinations [2]. But like breast and colorectal cancer, it has preventive screening recommendations, which not only can identify cancer but also detect precancerous tissue that can be removed. Because of early cancer detection, preventive cancer screenings can reduce mortality. For example, a recent study found that if cancer screenings increased by 10%, it would result in a reduction of deaths by nearly 1800 persons for breast cancer, over 11,000 persons for colorectal cancer, and over 1,700 persons for cervical cancer [3]. The USPSTF is one of the guiding scientific bodies on screening recommendations. Currently, routine screening for cervical and colorectal cancers has a grade “A” recommendation [4, 5], while breast cancer screening has a grade “B” recommendation [6].

Despite longstanding national guidelines recommending screening, screening uptake disparities continue to persist. Notably, the uptake of cancer screening in foreign-born populations in the U.S. is less than that in the native-born population. A cross-sectional survey found that foreign-born respondents who identified as White, Hispanic, or Asian-American and Pacific Islander (AAPI) were significantly less likely than U.S.-born adults who identified as White to report Pap smear uptake [7], which, on its own, in addition to HPV co-testing or with HPV testing alone, is the current recommendation for cervical cancer screening [4]. Similar results were found for foreign-born individuals who identified as Hispanics and AAPI when assessing the uptake of colorectal screening methods, including fecal occult blood testing and sigmoidoscopy, and breast cancer screening with mammography [7]. Additionally, data from the National Health Interview Survey from 2000 to 2010 comparing breast, cervical, and colorectal screening uptake among U.S.-born citizens, foreign-born citizens, and foreign-born non-citizens found that U.S.- and foreign-born citizens have similar rates of screening while foreign-born noncitizens have lower rates of screening [8]. This illustrates the disparity among the foreign-born population and the need to understand barriers to cancer screening within this population.

Akin to the definition developed in the 1951 Refugee Convention [9], the U.S. defines a refugee as an individual “who is unable or unwilling to return to [his, her, or their] country of nationality because of persecution or a well-founded fear of persecution on account of race, religion, nationality, membership in a particular social group, or political opinion” [10]. In contrast, immigrants encompass individuals who choose to move to another country for reasons outside of persecution without impediments to returning to their home country [11]. Aside from obtaining asylum in the U.S., the legal status of a refugee provides individuals with eight months of healthcare coverage and qualifies them to apply for health insurance under the Patient Protection and Affordable Care Act [12]. The number of newcomer refugees varies annually, but in 2022, there were over 25,000 refugees admitted to the U.S., and the number has ranged from about 22,000 to 84,000 annually over the past ten years [13].

Systematic reviews have assessed barriers to cancer screening for breast, cervical, and colorectal cancer among immigrant populations in the U.S., confirming lower cancer screening rates for foreign-born than native-born people [14–18]. These reviews identified barriers impacting immigrants at large, including lack of health insurance, language barriers, and limited understanding of cancer screenings, and barriers unique to specific immigrant populations, including fatalistic views derived from religious beliefs, familial influences, a lack of perceived susceptibility to cancer, and cultural gender norms, among others [14–18]. Additionally, barriers unique to specific ethnic and cultural groups were noted, including a) the role of childbirth, sexual activity, menses, and stress in cancer susceptibility for individuals who identify as Hispanic, b) administrative processes to establish healthcare for individuals who identify as African Americans, and c) stigmatization by community members and providers for Asian immigrants [14].

Despite the extensive characterization of barriers to cancer screening for breast, cervical, and colorectal cancers in the U.S. [19], current reviews assessing cancer screening barriers do not distinguish between immigrant and refugee populations and primarily cover breast and cervical cancer screenings [14–17]. We located one systematic review that focused on refugee women from Muslim-majority countries living in the U.S., which reported lower adherence rates relative to other U.S. women and identified barriers at the intrapersonal, interpersonal, institutional, and community levels [18]. But shortcomings of these findings were that they did not include colorectal screening rates and barriers for male refugees, they focused on only one sub-set of refugees (from Muslim-majority countries), and they selected studies that had samples from high refugee-producing countries rather than selecting all studies with refugee status as the sampling requirement.

The purpose of this rapid review is to identify barriers to cancer screening for breast, cervical, and colorectal cancers among a diverse set of refugee populations resettled in the U.S. By distinguishing refugees from the broader classification of immigrants, this review aims to assess the complexities in accessing and utilizing cancer screening services unique to refugee communities.

## Methods

### Objective

Given that non-refugee immigrants and refugees are often collectively assessed under the context of “immigrants,” little distinction is afforded to the challenges refugees face in various facets of life in the U.S., including preventive healthcare measures like cancer screenings. Thus, it is important to distinguish cancer screening barriers faced by refugee communities to inform public health interventions focused on increasing cancer screening rates in this vulnerable population. The research question guiding this review is as follows: What are barriers to breast, cervical, and colorectal cancer screenings experienced by refugees resettled in the U.S.?

### Eligibility Criteria

The eligibility criteria established for this rapid review are (1) studies written in the English language, (2) qualitative or observational studies, (3) studies based in the U.S., (4) studies that specify the sample as refugees, (5) studies focused on elucidating cancer screening barriers for breast, cervical, or colorectal cancer, and (6) studies published in the year 2010 or later. The time frame was selected to ensure focus on the most recent literature. The exclusion criteria established for this review include (1) studies focused only on non-refugee immigrant populations, (2) studies that do not distinguish between immigrants and refugees, and (3) interventional studies. Given the review’s focus on highlighting unique and nuanced barriers perceived by refugees or related to refugee status and experiences rather than identifying unrecognized barriers in the setting of interventions, interventional studies were excluded from the review.

### Information Sources

The PubMed and Scopus databases were employed to conduct the search strategies for this rapid review. These databases were selected due to the extensive variety and volume of literature covered, including literature relevant to healthcare services and minority populations. The PubMed and Scopus database searches were conducted on March 4th, 2024.

### Search Strategies

With the assistance of the research librarians at Thomas Jefferson University, a comprehensive list of key terms and Medical Subject Headings (MeSH) was developed for the PubMed and Scopus database searches. The search was restricted to “Title/Abstract” to limit the retrieval of irrelevant literature. Selected search terms for the population of interest include “refugees,” “asylum seekers,” and “immigrants.” Although literature focused on non-refugee immigrant populations is excluded from the review, the term “immigrants” was included in the search to retrieve relevant literature on elucidating cancer screening barriers for refugee and immigrant communities. A broad selection of terms related to barriers was incorporated to cover all potentially significant cancer screening barriers sufficiently. Such terms include “barriers,” “culture,” health beliefs,” “limited English proficiency,” “communication barriers,” “socioeconomic factors,” “mental health,” “psychological trauma,” and others. Terms focused on the outcome of interest include “cancer screening,” “early detection of cancer,” and terms related to breast, cervical, and colorectal cancer screenings. The literature identified in the database searches was imported to RefWorks and processed by two reviewers independently. See Supplemental Table 1 for the complete search strategy.

### Study Selection Process

The study selection process implemented the guidelines outlined by the Preferred Reporting Items for Systematic Reviews and Meta-analyses (PRISMA) [20]. Deduplicated literature was screened by title and abstract for evidence of the eligibility criteria. Studies that passed the initial review were then re-screened via a full-text review to identify the literature most relevant to the research question. Any discrepancies in the final selection of studies were discussed and resolved among the two reviewers.

### Data Collection Process and Data Items

Data from the final selection of studies was extracted and organized using Microsoft Excel. Preliminary data extraction from each study included the authors’ names, the title, and the year of publication. Specific data items collected from each study were as follows: study location, study design (qualitative, mixed-method, or observational), ethnicity or country-of-origin of refugee population(s) assessed, sample demographics (sex, age, education, years in the U.S., years in a refugee camp or second country, English fluency, employment status, marital status, religion, and health insurance status), sample size, method(s) of data collection, cancer screening rates of the study sample, and identified barriers for breast, cervical, or colorectal cancer screenings.

### Outcomes and Data Synthesis

The primary outcome selected for analysis from each study was the identification of barriers to breast, cervical, or colorectal cancer screenings. Barriers were defined as factors perceived by individuals in the study populations as preventing access to appropriate services for these cancer screenings. Data on barriers, collected through focus groups, interviews, and surveys, were extracted from each study and reviewed to uncover recurring themes identified by the authors of the included studies. The extracted data were then organized under these themes.

## Results

### Studies Identified for Review

**Fig. 1:**
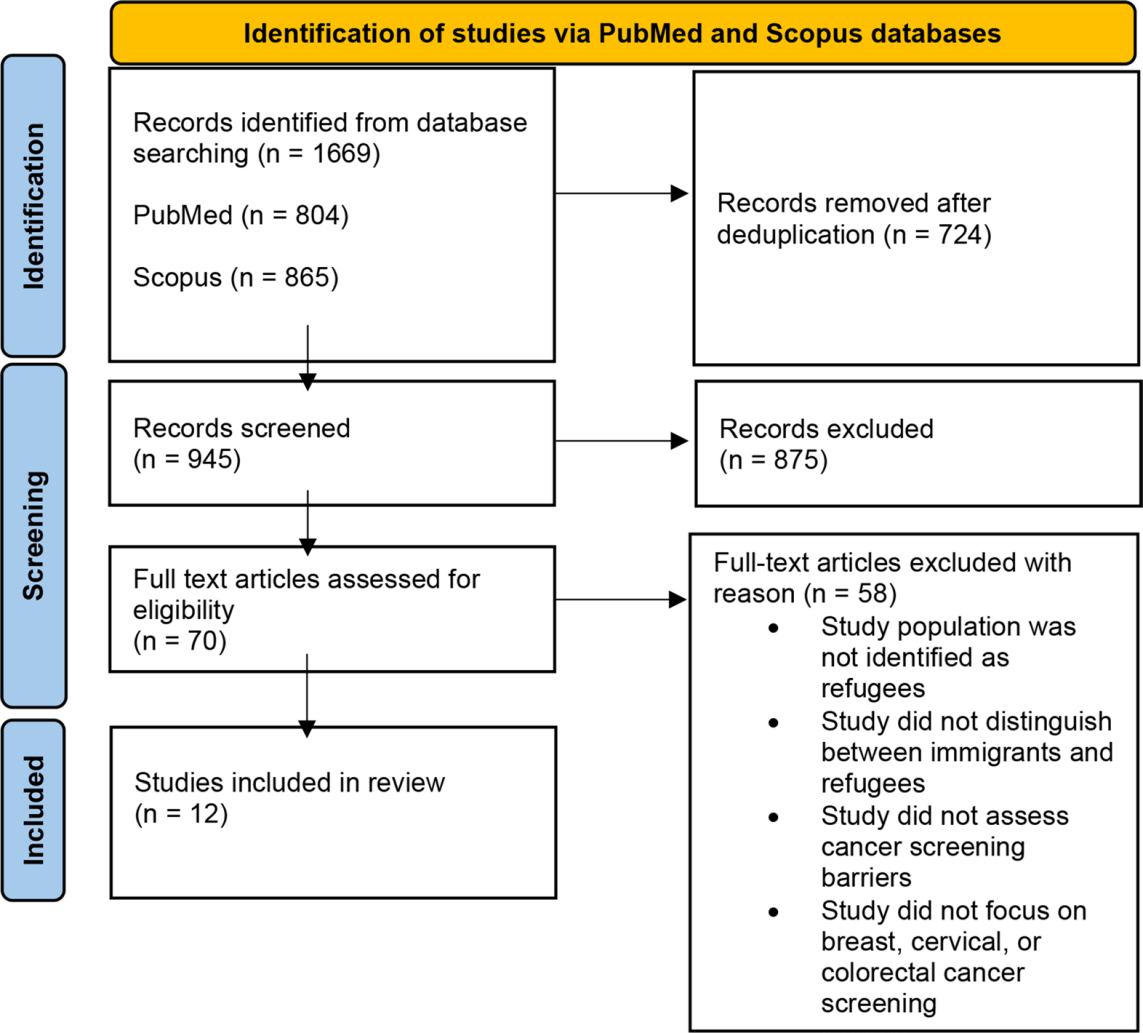
PRISMA Flow Diagram of Study Selection Process

After conducting the outlined search strategy, 1669 articles (804 from the PubMed database and 865 from the Scopus database) were retrieved and imported to RefWorks (see Fig. 1). The articles were merged, and 724 duplicate articles were removed. Of the 945 articles screened for relevance via a title/abstract review, 875 were excluded. Seventy eligible articles were screened via a full-text review, and 12 were included in this rapid review. The included articles sampled refugee-specific communities and assessed barriers to breast, cervical, or colorectal cancer screenings.

### Characteristics of Selected Studies

Of the twelve studies, eight provided qualitative data through focus groups or interviews [21–28], two provided observational data through cross-sectional surveys [23, 29], and two provided both qualitative and observational data [30, 31] (see Table 1). All but one included study [31] employed non-probability sampling methods for sample recruitment. The studies took place across various states, including Nebraska (*N* = 1), Ohio (*N* = 3), Washington (*N* = 1), Massachusetts (*N* = 2), Minnesota (*N* = 1), California (*N* = 2), New York (*N* = 1), and Michigan (*N* = 1) [21–32]. Six studies focused on a single ethnicity of refugees [21, 23, 25, 28, 30, 32], and six studies provided an analysis of multiple refugee ethnicities [22, 24, 26, 27, 31] or refugee and non-refugee immigrant communities [29]. Represented refugee communities included Afghani (*N* = 1), Asian (*N* = 1), Bhutanese (*N* = 3), Bosnian (*N* = 1), Burmese (*N* = 2), Iraqi (*N* = 2), Cambodian and Laos (*N* = 1), and Somali refugees (*N* = 3) [21, 23–30, 32]. One study assessed refugees from across the Middle East and Sub-Saharan Africa [22], while another assessed Arabic-speaking refugees but did not denote their country of origin [31]. Sample sizes for each refugee population ranged from 17 to 217. Refugee samples were primarily female, except for two studies that included male refugees [27, 29]. Nine studies addressed cervical cancer screening [21–24, 27, 29–32], five studies addressed breast cancer screening [25–29], and two studies addressed colorectal cancer screening [27, 29]. Screening rates ranged from 13.9% to 90% for cervical cancer [21, 23, 25, 27, 29–32], 0% to 65.9% for breast cancer [27–29], and 0% to 32% for colorectal cancer [27, 29]. See Supplemental Table 2 for an overview of the demographic data for each study sample.

**Table 1:** Key characteristics of included studies

Author	Setting	Study population (sample size)	Sampling method	Study design & analysis	Cancer Screening	Screening Rates
Haworth et al. [29]	Omaha, Nebraska	Bhutanese refugees (*N* = 69)	Nonprobability sampling	Mixed method: focus group & thematic analysis + cross-sectional survey	Cervical	**Pap Smear** (*N* = 36) Ever had a pap smear: 13.9% Within past year: 8.3% Within past 2 years: 5.6%
Kue et al. [23]	Columbus, Ohio	Bhutanese-Nepali refugees (*N* = 97)	Nonprobability sampling	Observational cross-sectional survey & logistic regression model	Cervical	**Pap smear** (*N* = 97) Ever had pap smear: 44.3%
Lor et al. [24]	King County, Washington	Bhutanese refugees (*N* = 31) Burmese refugees (*N* = 27)	Nonprobability sampling	Qualitative: focus group discussions & thematic analysis	Cervical	**Cervical cancer screening** *Burmese (N = 31*) Before U.S. resettlement: 29% After U.S. resettlement: 64% *Bhutanese (N = 27)* Before U.S. resettlement: 11% After U.S. resettlement: 44%
Allen et al. [21]	Minneapolis & St. Paul, Minnesota	Somali refugees (*N* = 31)	Nonprobability sampling	Qualitative: focus group discussions & thematic analysis	Cervical	**Pap Smear** (*N* = 31) Ever had a pap smear: 90% Within past year: 42% Within one to three years: 32% More than three years ago: 26%
Ghebrendrias et al. [22]	San Diego, California	Sudanese, Somalian, Kenyan, Ethiopian, Eritrean, Congolese, Ugandan, Syrian, Iraqi, Egyptian, and Moroccan refugees (*N* = 53)	Nonprobability sampling	Qualitative: Semi structured focus groups & thematic analysis	Cervical	Not reported
Kue et al. [32]	Franklin county, Ohio	Cambodian and Lao refugees (*N* = 22)	Nonprobability sampling	Qualitative: interviews & thematic analysis	Cervical	**Pap smear** (*N* = 22) *Mothers (N = 11)* Ever had a pap smear: 72.7% *Daughters (N = 11)* Ever had a pap smear: 90.9%
Al Abdul Kader et al. [31]	Cleveland, Ohio	Arabic speaking refugees (*N* = 20)	Random sampling	Mixed method: focus group & thematic analysis + cross-sectional survey	Cervical	**Pap Smear** (*N* = 20) Within past 5 years: 65% More than 5 years ago: 10%
Saadi et al. [25]	Massachusetts	Iraqi refugees (*N* = 20)	Nonprobability sampling	Qualitative: interviews & thematic analysis	Breast	Not reported
Saadi et al. [26]	Massachusetts	Bosnian refugees (*N* = 20) Somali refugees (*N* = 17) Iraqi refugees (*N* = 20)	Nonprobability sampling	Qualitative: interviews & thematic analysis	Breast	Not reported
Shirazi et al. [28]	Alameda County, California	Afghani refugees (*N* = 53)	Nonprobability sampling	Qualitative: interviews & thematic analysis	Breast	**Mammogram** Ever had a mammogram (*N* = 53): 65.9% Last mammogram over 2 years ago (*N* = 35, those who ever had mammogram): 50.1%
Schuster et al. [27]	Buffalo, New York	Somali Bantu refugees (*N* = 15) Karen refugees (*N* = 15)	Nonprobability sampling	Qualitative: interviews & thematic analysis	Breast, Cervical, Colorectal	**Cancer screening general** *Somali (N = 15)* In home country: 0% In U.S.: 27% *Karen (N = 15)* In home country: 0% In U.S.: 33% **Pap smear***(N* = 26, age eligible sample): 34.6% (4 Somali & 5 Karen) **Mammogram***(N* = 5, age eligible sample): 0% **Colorectal cancer screening** (*N* = 8, age eligible sample): 0%
Wu et al. [29]	Michigan	Asian refugees & non-refugees (*N* = 217)	Nonprobability sampling	Observational: cross-sectional survey & logistic regression model	Breast, Cervical, Colorectal	**Pap smear** (*N* = 114) Refugee: 62% Non-refugee: 73% **Mammogram** (*N* = 87)* Refugee: 60% Non-refugee: 73% (*p < 0.05) **Sigmoidoscopy** (*N* = 110)* Refugee: 25% Non-refugee: 67% (*p < 0.01) **Colonoscopy** (*N* = 110)* Refugee: 32% Non-refugee: 64% (*p < 0.01)

### Barriers to Cancer Screenings

We identified six key themes that encompass the barriers perceived by the refugee populations represented in the included studies. These themes are outlined below and summarized in Table 2.

**Table 2:** Identified cancer screening barriers among refugee populations

Author (year)	Study population	Language and Health System Navigation	Cost, Transportation, and Time	Health Knowledge and Exposure	Fear of Cancer Screening and Diagnosis	Cultural and Religious Beliefs	Refugee experience or status
Haworth et al. [30]	Bhutanese refugees	Language, the complexity of the healthcare system	Lack of insurance, transportation	Limited knowledge of cervical cancer, lack of trust with healthcare providers & interpreters	Fear of pain during a pap smear	Shyness, stigma	Experiences of sexual abuse by male health providers in refugee camps
Kue et al. [23]	Bhutanese refugees	None identified	Lack of time	None identified	Fear of pain during a pap smear	Shyness	None identified
Lor et al. [24]	Bhutanese refugees; Burmese refugees	Language, difficulties navigating the U.S. healthcare system	Cost, transportation	Lack of familiarity with preventive care, misconceptions about cause of cervical cancer, mistrust of reproductive healthcare	Fear of screening	Stigma and embarrassment around pap smear	Experiences with violence in refugee camps
Saadi et al. [25]	Iraqi refugees	None identified	Insurance, transportation	None identified	Fear of pain during a mammogram, fear of cancer diagnosis	Modesty	Experiences with war and death in home country
Saadi et al. [26]	Bosnian refugees; Somali refugees; Iraqi refugees	Navigating the appointment system	Lack of time resulting from work and childcare	Lack of exposure with physicians in home country, mistrust of healthcare providers	Fear of pain during a mammogram, fear of cancer diagnosis	Fatalism, modesty	Experiences with war and death in home country
Shirazi et al. [28]	Afghani refugees	Communication barriers, lack of familiarity with screening locations and scheduling screenings	Lack of insurance, transportation	Lack of understanding of preventive healthcare	None identified	Modesty concerns, patriarchal decision making	None identified
Schuster et al. [27]	Somali Bantu refugees; Burmese Karen refugees	Language, the complexity of navigating the healthcare system, difficulties in scheduling appointments	Transportation	Low health literacy	None identified	Modesty concerns, patriarchal decision making, fatalism	Survival in refugee camps
Wu et al. [29]	Asian refugees & non-refugees	None identified	None identified	None identified	None identified	None identified	Non-refugee Asians were 24 times more likely [95% CI: 2.68,209.56] to have ever received a mammogram than Asian refugees. Non-refugee Asians were 5 times more likely to have received a colonoscopy than Asian refugees [95% CI: 1.64,15.90]
Allen et al. [21]	Somali refugees	None identified	None identified	Misconceptions about the cause of cervical cancer, limited knowledge of when Pap smear should be completed	None identified	None identified	None identified
Ghebrendrias et al. [22]	Middle Eastern & Sub-Saharan African refugees	Language	None identified	Limited knowledge of cervical cancer and its causes	None identified	Negative perceptions of cervical exam and pap smear, modesty concerns, religious stereotypes among providers, shame around discussion of women’s health issues	Limited to no screening opportunities in refugee camps
Kue et al. [32]	Cambodian & Laos refugees	Language	None identified	Limited knowledge of cervical cancer and its causes	None identified	Limited discussion of sexual and reproductive health due to cultural taboos and embarrassment	Limited discussion of and tendency to repress memories of their experiences as refugees
Al Abdul Kader et al. [31]	Arabic speaking refugees	Language	Lack of insurance, transportation, time	Lack of knowledge of cervical cancer and pap smear	Fear of cancer, anticipated pain/discomfort during pelvic exam	Possibility of encountering a male physician	None identified

#### Language and Health System Navigation

Seven studies reported language as a barrier to either breast, cervical, or colorectal cancer screenings [22, 24, 27, 28, 30–32], while five studies noted difficulties in navigating the U.S. healthcare system [24, 26–28, 30] (see Table 2). Afghani refugees reported significant challenges with these barriers in obtaining breast cancer screenings, where 75% of the interviewed individuals noted a lack of interpreters, language difficulties, and issues in scheduling appointments as prominent barriers [28]. Only language was noted as a barrier by Cambodian and Laos refugees [32] and refugees from the Middle East and Sub-Saharan Africa [22]. In a comparative analysis of cancer screening barriers for Somali Bantu and Burmese Karen refugees, only Karen refugees noted the process of scheduling a screening and finding the screening location as prominent barriers [27].

### Cost, Transportation, and Time

Five studies reported cost or lack of insurance coverage as a barrier to cancer screening [24, 25, 28, 30, 31], and six reported transportation as a barrier [24, 25, 27, 28, 30, 31]. However, for Iraqi refugees interviewed in Saadi et al. [25], insurance coverage and transportation were among the least reported barriers, with a refugee noting, “Oh, [transportation is] so easy. In the summer, the shuttles and hospitals are kept cold, and in the winter, they’re kept warm, and we enjoy riding on a bus, going out [25].” It is important to note that the sample interviewed in this study were all insured under the Massachusetts universal healthcare regulations. Three studies reported lack of time as a barrier [23, 26, 31], with one citing work and childcare commitments specifically as a limitation to obtaining cancer screening among 21% (12/57) of interviewed Somali, Bosnian, and Iraqi refugees [26].

### Health Knowledge and Exposure

Nine studies noted limited health knowledge of or exposure to cancer, cancer screening, or preventive healthcare as barriers to breast, cervical, and colorectal cancer screenings [21, 22, 24, 26–28, 30–32]. A majority of Bhutanese refugees in Haworth et al. [30] had never heard of cervical cancer or a pap smear. Likewise, a majority of Afghan refugees in Shirazi et al. [28] demonstrated a very low level of knowledge of breast cancer and screening. Burmese and Bhutanese refugee women in Lor et al. [24] expressed misconceptions about the etiology of cervical cancer, with some stating the cancer was related to personal hygiene and cleanliness. Some Somali refugee women also expressed misconceptions related to cervical cancer, including genetic predisposition and birth control as risk factors, while others correctly noted the role of infection and sexual activity in increasing risk [21]. Utilizing healthcare services only when symptomatic was expressed by Somali Bantu and Karen refugees [27] and Burmese and Bhutanese refugees [24]. In one study, 76% of interviewed Somali women reported a lack of exposure to doctors in Somalia, while almost all Bosnian and Iraqi refugees interviewed in the study had been exposed to doctors before resettling in the U.S. [26]. One of the Somali women reported that her community would hide from international health workers conducting vaccinations in Somalia due to uncertainty about what they were providing. Mistrust in healthcare providers was also expressed by Bhutanese and Burmese refugees [24, 30].

### Fear of Cancer Screening and Diagnosis

Six studies reported fear of the screening experience as a perceived barrier to cervical and breast cancer screening [23–26, 30, 31]. One study’s survey revealed that 85.7% of the Bhutanese refugees feared a pap smear was painful [30]. Likewise, cervical cancer screening rates were found to be lower among Bhutanese refugees who reported fear of having a Pap smear (44.2% vs. 68.0%, *p* = 0.01) [23]. However, the screening rate was significantly higher among Bhutanese refugees who viewed Pap smears as uncomfortable or painful (83.3% vs. 41.7%, *p* = 0.001). For breast cancer screening, Iraqi, Somali, and Bosnian refugees reported not only perceived fear of pain during mammograms as a barrier to screening but also fear of receiving a cancer diagnosis [25, 26]. Similarly, fear of cancer was the most reported concern for cervical cancer screening among Arabic-speaking refugees [31].

### Cultural and Religious Beliefs

Nine studies noted modesty, shyness, and stigma as barriers to breast and cervical cancer screening [22–28, 30, 32]. In one study, about one third of Bosnian, Iraqi, and Somali refugees cited modesty as a deterrent to breast cancer screening; however, this concern was not viewed as an absolute barrier as some of the women also mentioned religious beliefs prioritizing individual health over principles of modesty [26]. Several women also expressed a strong preference for female providers to administer cancer screenings [23, 25, 28]. Arabic-speaking refugees interviewed by Al Abdul Kader et al. [31] expressed significant concern with gynecologic care and pap smears delivered by male providers in both the outpatient setting and in emergency situations, with some reporting refusal of high acuity care from male providers.

Fatalistic perspectives towards cancer among predominantly Muslim communities were noted for Somali, Iraqi, and Bosnian refugees [26, 27]. However, religious fatalism was not reported as a barrier by Muslim Afghani refugees [28]. Rather, Afghani women suggested that Islamic beliefs required a proactive approach to healthcare. Patriarchal hierarchy in health decisions, including around breast cancer screening, was noted as a barrier by Afghani refugees, where 90% of Afghani refugees interviewed reported their dependence on male partners for making and traveling to an appointment [28]. Discomfort with religious stereotypes among providers towards Muslims was noted as a barrier to cervical cancer screening by Middle Eastern and Sub-Saharan African refugees [22].

### Refugee Experience or Status

Eight studies relayed barriers associated with refugee experiences (i.e., war, political violence, displacement/migration process, and lack of resources) [22, 24–27, 30, 32] or refugee status [29] (see Table 2). Bhutanese refugees described experiences of abuse and sexual assault by male health providers while in refugee camps in Nepal and India; these experiences resulted in an almost unanimous preference for female health providers when receiving cervical cancer screenings [30]. Both Burmese and Bhutanese refugees reported that their experiences with the death of family members and violence while migrating to refugee camps led them to undermine the importance of cervical cancer screenings [24]. Iraqi refugees noted similar sentiments, where breast cancer screening was perceived as irrelevant compared to the immediate threat of death brought about by war and violence [25]. A comparative analysis of Iraqi, Bosnian, and Somali refugees revealed that the impact of war on breast cancer screening was not as central to Bosnian and Somali refugees as it was to Iraqi refugees [26]. An analysis of barriers for Somali Bantu and Burmese Karen refugees also highlighted the theme of survival in refugee camps [27]. Although the impact of Cambodian and Laos refugee experiences on cervical cancer screening was not directly discussed, mothers and daughters in the study noted significant hesitation on the part of the mothers to discuss their refugee experiences with their daughters and others alike, as many of their memories were too painful to revisit [32].

One study provided a unique analysis of the role of refugee status in predicting cancer screening rates for breast, cervical, and colorectal cancers among Asian Americans [29]. Logistic regression models were calculated to predict the odds of receiving a mammogram and colonoscopy for the predictor variables of refugee status, education level, insurance coverage, having a regular doctor, and years in the U.S. Refugee status was determined to be the strongest predictor for receiving a mammogram, where non-refugee Asians were 24 times more likely [95% CI: 2.68, 209.56] to have ever received a mammogram than Asian refugees. Non-refugee Asians were also five times more likely to have received a colonoscopy than Asian refugees [95% CI: 1.64, 15.90].

## Discussion

Cancer screening disparities between foreign and native-born populations in the U.S. have been extensively documented. Yet, little attention has been given to cancer screening barriers for refugee communities. This rapid review identified various barriers to breast, cervical, and colorectal cancer screening among an ethnically diverse range of refugee populations resettled in the U.S. Most of the included studies assessed challenges associated with language, healthcare system navigation, insurance coverage or cost, transportation, and time. The variations in the perception of these barriers among different ethnicities may reflect inherent differences in their experiences adapting to life in the U.S., stemming from the varied resources available for these communities in their resettlement locations. Health knowledge, exposure, and beliefs as well as cultural and religious norms, were consistently identified across most studies; however, specific barriers also varied based on home country of origin and religious identity. These barriers encompassed a lack of knowledge, misconceptions, or fear around cancer and screening, gender and modesty norms, and patriarchal family structures, among others. Regarding the role of the refugee experience, a majority of the studies highlighted the impact of war, violence, sexual assault, and trauma in decreasing a refugee’s concern for preventable disease in the wake of life-threatening experiences. Although trauma is not limited to the refugee experience, the distinction of a refugee as an individual fleeing persecution or death from political violence and war carries a burden of trauma unique to this population. Thus, it is essential to distinguish between refugee and non-refugee immigrant groups to avoid overshadowing or generalizing the impact of the refugee experience on health behaviors. Very few studies touched on barriers to colorectal cancer screening and perceived barriers for men. Although colorectal cancer screening was the only cancer screening included relevant to men, the lack of research among this cohort of refugees points to a critical gap in the literature, especially considering the recent change in colorectal cancer screening guidelines recommending screening at a younger age.

### Solutions to Barriers

The findings of this review suggest the need for culturally tailored interventions that address the unique barriers perceived by different ethnic groups of refugees. The current literature provides insight into the success of such interventions in increasing the intent to receive and rates of cancer screenings among refugees.

### EducationInterventions to Address Health Knowledge and Exposure and Fear of Screening and Diagnosis

A pilot video intervention was conducted to raise awareness of and intention to receive cervical cancer screening among a small sample of Burmese and Bhutanese refugees [33]. The researchers developed culturally tailored videos that employed storylines and cultural norms specific to each ethnic group of refugees. They found that both Bhutanese-Nepali and Karen Burmese women were significantly more aware of cervical cancer screening, and Bhutanese-Nepali women were more likely to be screened for cervical cancer. This provides evidence for culturally tailored narratives and educational videos and offers a scalable intervention to address the barriers refugees experience in obtaining cancer screening.

Another study developed a program centered on community-academic partnerships to promote and deliver breast health education, which resulted in 80% of the participants obtaining a mammogram [34]. Support from respected leaders within communities led to the engagement of individual community members and can be used as a model to build effective and sustainable programs. The authors described how expanding into topics of interest for the community is essential to long-term engagement, specifically highlighting the need for education on colonoscopies and pap smears within immigrant and refugee communities. By establishing long-term partnerships, it is possible to enhance cancer screening of varying types and address other health inequities in the community.

### Language and Health System Navigation Interventions

Culturally tailored patient navigation interventions and programs demonstrate the effectiveness of targeted support in addressing healthcare barriers for refugees. For one, the Refugee Health Navigator Program at Georgetown University pairs interdisciplinary student volunteers with newly arrived families in the D.C. region to aid them in navigating the U.S. healthcare system, including overcoming language barriers, obtaining access to and understanding insurance, and finding appropriate healthcare providers for their needs [35]. Similarly, a tailored patient navigation program for Somali, Bosnian, and Iraqi refugees provided personalized guidance with appointment scheduling, transportation, and insurance issues in addition to addressing cultural barriers unique to different ethnicities [36]. Breast cancer screening uptake increased from 64.1% (95% CI: 4–77%) to 8.2% (95% CI: 7–88%) three years after program initiation. This increased screening rate was similar to a cohort of English-speaking (80.0%, 95% CI: 7–86%, *p* = 0.3) and Spanish-speaking (82.8%, 95% CI: 7–88%, *p* = 0.7) women from the same health center. A follow-up study assessing the program’s long-term impact found that four years after the original patient navigation intervention concluded, screening rates among refugee women decreased but were not statistically different from English-speaking women and remained well above the screening rates prior to the intervention [37]. Although a long-term patient navigation program may not be feasible given funding and resource constraints, this intervention provides evidence of the relatively sustained impacts of short-term patient navigation services on cancer screening uptake for refugees.

While not focused on refugee populations, other studies have assessed the utility of self-collected samples for cervical and colorectal cancer screenings, further addressing access barriers, including appointment scheduling, transportation, and time, as well as cultural norms like modesty concerns. A randomized control trial assessed whether mailing fecal occult blood testing (FOBT) kits directly to patients overdue for colorectal cancer screening improved screening rates among underserved populations compared to individuals receiving “usual care,” consisting of referrals made during regular clinician visits [38]. Among 104 patients who received the outreach intervention, 30% completed screening, compared to 5% in the usual care group (*p* < 0.001), with most screenings completed via FOBT. Cervical cancer screening with HPV self-testing kits in the healthcare setting was recently approved by the Food and Drug Administration to increase cancer screening access [39]. A randomized control trial among Somali immigrant women prior to approval found that Somali women offered a home-based HPV self-sampling test were 14 times more likely to complete screening within three months compared to those offered a clinic-based Pap test (*p* = 0.0002). These findings suggest the utility of self-administered and at-home screening tests in increasing the uptake of cancer screenings, particularly among vulnerable populations.

### Culturally and Religiously Tailored Interventions

Addressing religious beliefs alongside cultural norms has proven effective in increasing knowledge and intention to undergo cancer screening among underserved populations in the U.S. A workshop to address religious barriers to breast and cervical cancer screening among Somali American Muslim women and male imams demonstrated increased knowledge of the benefits of screening and intention to get a mammogram [40]. Both Somali American Muslim women and male imams showed increased disagreement with the statement “having breast or cervical cancer screening would be an immodest thing to do.” Similarly, a mosque-based, peer-led educational program for South Asian and Arab Muslim women was designed to increase mammography knowledge, decrease endorsement of barriers beliefs, and increase endorsement facilitator beliefs to mammography. The study found a statistically significant difference in mammography knowledge post-intervention (*p* = 0.0002), a trend towards statistical significance for aggregate facilitator beliefs (*p* = 0.08), and no significant changes with aggregate barrier beliefs. Despite this, the study did note a statistically significant decrease in the number of positive responses to the statement, “Breast cancer screening is not important because God decides who will get cancer” (*p* = 0.03), suggesting the potential of religiously tailored messages to address barrier health beliefs.

### Implications

This review’s findings also have implications for public health research and practice addressing cancer screening barriers for refugees resettled in the U.S. First, this review highlights the need to distinguish between immigrant and refugee communities in cancer screening and preventive health research. Refugees are a unique category of migrants granted a status separate from that of immigrants. Using the terms interchangeably may dilute the meaning of “refugee” and fail to address experiences unique to refugees that can impact their use of preventive health services and interactions with the U.S. healthcare system.

Additionally, the findings suggest the need to consider the role of trauma as a refugee experience barrier to cancer screening. Understanding the impact of trauma can aid in the development of interventions to increase screening that sufficiently addresses this barrier. In the context of refugees, trauma-informed care encompasses the understanding of trauma as a central aspect of a refugee community’s experience and the need to address trauma at all levels of care. One study assessed the implementation of cross-cultural trauma-informed care training interventions to improve mental healthcare for refugees administered by resettlement agency providers [41]. The training incorporated knowledge-based components to increase providers’ awareness of refugee trauma, mental health issues, and cultural expressions of distress and skill-based elements to increase providers’ listening skills, intervention capacity, and approaches to relaying self-care and mindfulness strategies. The intervention resulted in significant improvements in providers’ knowledge and skills in the realm of cross-cultural trauma-informed care, with an average change in the total core competency test score of 8.35 post-intervention (p < 0.001). Adapting such a training program for public health practitioners and healthcare providers can help refugee communities process internalized barriers to cancer screenings resulting from trauma and increase their desire to receive these screenings.

### Strengths and Limitations

This rapid review has several strengths. The terms included in the database searches were developed in consultation with a research librarian. The study selection process followed the guidelines outlined by PRISMA [19] to ensure the reproducibility of the methods employed. The article selection, data collection, and analysis were conducted by two reviewers, increasing internal validity. This review also included studies representative of several ethnic groups of refugees and various locations across the U.S., increasing the generalizability of the results.

There are some limitations to this review. Only two databases were searched for relevant literature. The review aimed to cover breast, cervical, and colorectal cancer screening barriers for men and women; however, colorectal cancer screening (*N* = 2) and samples of refugee men (*N* = 2) were underrepresented in the literature. Future research is needed to assess colorectal screening barriers faced by refugee communities resettled in the U.S. Additionally, because of the use of the terms “immigrant” and “refugee” interchangeably in the literature, some studies addressing cancer screening barriers faced by refugees may have been excluded due to the lack of transparency. When possible, future research should attempt to clearly define refugee communities apart from the broader category of immigrants so that differences between refugee and immigrant groups can be ascertained.

## Conclusion

Low screening rates for breast, cervical, and colorectal cancer screening among refugee communities resettled in the U.S. is a public health issue of critical importance. Increasing refugee cancer screening rates in the U.S. is imperative to improving their health and overall quality of life in the U.S. Although the literature has thoroughly identified barriers to breast, cervical, and colorectal cancer screening in the general population, there is a lack of attention to the barriers experienced by refugee groups. Among other findings, this rapid review identified variations in the barriers experienced by different ethnic groups of refugees and the impact of refugee experiences and trauma in shaping their perspectives on cancer screening. Thus, interventions developed to address the disparities in cancer screening seen among refugees must consider each ethnic community’s perceived barriers to screening and the impact of experiences associated with refugee status, including trauma. Future research is still needed to examine the effect of refugee-related experiences on the cancer screening perspectives of refugees more thoroughly.

## Electronic Supplementary Material

Below is the link to the electronic supplementary material.

Supplementary Material 1

Supplementary Material 2

## Data Availability

No datasets were generated or analysed during the current study.
