# Supplementary material for: Barriers to Breast, Cervical, and Colorectal Cancer Screenings Faced by Refugees Resettled in the United States: A Rapid Review
Source: J Immigr Minor Health. 2025 May 28;27(4):609–22. doi: 10.1007/s10903-025-01690-1 (PMC12255539; doi:10.1007/s10903-025-01690-1)
Supplement: Supplementary file 1 — Supplementary Material 1 [file 10903_2025_1690_MOESM1_ESM.docx]

**Supplemental Table 1**. Search strategies utilized on PubMed and Scopus databases on March 4^th^, 2024.

| Database | Search | Hits |
| --- | --- | --- |
| PubMed | (("refugee*"[Title/Abstract] OR "Refugees"[MeSH Terms] OR "migrant*"[Title/Abstract] OR "Transients and Migrants"[Title/Abstract] OR "Transients and Migrants"[MeSH Terms] OR "asylum seeker*"[Title/Abstract] OR "asylum"[Title/Abstract] OR "Emigrants and Immigrants"[Title/Abstract] OR "Emigrants and Immigrants"[MeSH Terms] OR "immigrant*"[Title/Abstract]) AND ("barrier*"[Title/Abstract] OR "perceived barrier*"[Title/Abstract] OR "culture*"[Title/Abstract] OR "Culture"[MeSH Terms] OR "Acculturation"[Title/Abstract] OR "Acculturation"[MeSH Terms] OR "cultural belief*"[Title/Abstract] OR "health belief*"[Title/Abstract] OR "Awareness"[Title/Abstract] OR "Awareness"[MeSH Terms] OR "health knowledge attitudes practice"[Title/Abstract] OR "health knowledge, attitudes, practice"[MeSH Terms] OR "Health Services Accessibility"[Title/Abstract] OR "Health Services Accessibility"[MeSH Terms] OR "health facility environment*"[Title/Abstract] OR "Health Facility Environment"[MeSH Terms] OR "Patient Acceptance of Health Care"[Title/Abstract] OR "Patient Acceptance of Health Care"[MeSH Terms] OR "limited English proficiency"[Title/Abstract] OR "limited English proficiency"[MeSH Terms] OR "communication barrier*"[Title/Abstract] OR "Communication Barriers"[MeSH Terms] OR "socioeconomic factor*"[Title/Abstract] OR "Socioeconomic Factors"[MeSH Terms] OR "Mental Health"[Title/Abstract] OR "Mental Health"[MeSH Terms] OR "trauma and stressor related disorder*"[Title/Abstract] OR "Trauma and Stressor Related Disorders"[MeSH Terms] OR "stress disorders post traumatic"[Title/Abstract] OR "stress disorders, post traumatic"[MeSH Terms] OR "PTSD"[Title/Abstract] OR "psychological trauma*"[Title/Abstract] OR "Psychological Trauma"[MeSH Terms] OR "stigma*"[Title/Abstract]) AND ("cancer screening*"[Title/Abstract] OR "early detection of cancer*"[Title/Abstract] OR "Early Detection of Cancer"[MeSH Terms] OR "breast cancer"[Title/Abstract] OR "breast neoplasm*"[Title/Abstract] OR "Breast Neoplasms"[MeSH Terms] OR "mammogram*"[Title/Abstract] OR "mammography*"[Title/Abstract] OR "Mammography"[MeSH Terms] OR "cervical cancer"[Title/Abstract] OR "uterine cervical neoplasm*"[Title/Abstract] OR "Uterine Cervical Neoplasms"[MeSH Terms] OR "pap test*"[Title/Abstract] OR "Papanicolaou test*"[Title/Abstract] OR "Papanicolaou Test"[MeSH Terms] OR "pap smear*"[Title/Abstract] OR "HPV"[Title/Abstract] OR "colon cancer"[Title/Abstract] OR "colonic neoplasm*"[Title/Abstract] OR "Colonic Neoplasms"[MeSH Terms] OR "colonoscopy*"[Title/Abstract] OR "Colonoscopy"[MeSH Terms])) AND (2010:2024[pdat]) | 804 |
| Scopus | ( ( TITLE-ABS-KEY ( "refugee*" ) )  OR  ( TITLE-ABS-KEY ( "migrant*" ) )  OR  ( TITLE-ABS-KEY ( "transients and migrants" ) )  OR  ( TITLE-ABS-KEY ( "asylum seeker*" ) )  OR  ( TITLE-ABS-KEY ( "asylum" ) )  OR  ( TITLE-ABS-KEY ( "emigrants and immigrants" ) )  OR  ( TITLE-ABS-KEY ( "immigrant*" ) ) )  AND  ( ( TITLE-ABS-KEY ( "barrier*" ) )  OR  ( TITLE-ABS-KEY ( "perceived barrier*" ) )  OR  ( TITLE-ABS-KEY ( "culture*" ) )  OR  ( TITLE-ABS-KEY ( "acculturation" ) )  OR  ( TITLE-ABS-KEY ( "cultural belief*" ) )  OR  ( TITLE-ABS-KEY ( "health belief*" ) ) OR  ( TITLE-ABS-KEY ( "awareness" ) )  OR  ( TITLE-ABS-KEY ( "health knowledge” ) )  OR  ( TITLE-ABS-KEY ( "health attitude*” ) ) OR  ( TITLE-ABS-KEY ( "health practice*” ) ) OR  ( TITLE-ABS-KEY ( "health services accessibility" ) )  OR  ( TITLE-ABS-KEY ( "health facility environment*" ) )  OR  ( TITLE-ABS-KEY ( "patient acceptance of health care" ) )  OR  ( TITLE-ABS-KEY ( "limited English proficiency" ) )  OR  ( TITLE-ABS-KEY ( "communication barrier*" ) )  OR  ( TITLE-ABS-KEY ( "socioeconomic factor*" ) )  OR  ( TITLE-ABS-KEY ( "mental health" ) )  OR  ( TITLE-ABS-KEY ( "trauma and stressor related disorder*" ) )  OR  ( TITLE-ABS-KEY ( "stress disorders, post-traumatic" ) )  OR  ( TITLE-ABS-KEY ( "PTSD" ) )  OR  ( TITLE-ABS-KEY ( "psychological trauma*" ) )  OR  ( TITLE-ABS-KEY ( "stigma*" ) ) )  AND  ( ( TITLE-ABS-KEY ( "cancer screening*" ) )  OR  ( TITLE-ABS-KEY ( "early detection of cancer" ) )  OR  ( TITLE-ABS-KEY ( "breast cancer" ) )  OR  ( TITLE-ABS-KEY ( "breast neoplasm*" ) )  OR  ( TITLE-ABS-KEY ( "mammogram*" ) )  OR  ( TITLE-ABS-KEY ( "mammography*" ) )  OR  ( TITLE-ABS-KEY ( "cervical cancer" ) )  OR  ( TITLE-ABS-KEY ( "uterine cervical neoplasm*" ) )  OR  ( TITLE-ABS-KEY ( "pap test*" ) )  OR  ( TITLE-ABS-KEY ( "Papanicolaou test*" ) )  OR  ( TITLE-ABS-KEY ( "pap smear*" ) )  OR  ( TITLE-ABS-KEY ( "HPV" ) )  OR  ( TITLE-ABS-KEY ( "colon cancer" ) )  OR  ( TITLE-ABS-KEY ( "colonic neoplasm*" ) )  OR  ( TITLE-ABS-KEY ( "colonoscopy*" ) ) )  AND  ( LIMIT-TO ( PUBYEAR ,  2024 ) OR LIMIT-TO ( PUBYEAR ,  2023 ) OR LIMIT-TO ( PUBYEAR ,  2022 ) OR LIMIT-TO ( PUBYEAR ,  2021 )  OR  LIMIT-TO ( PUBYEAR ,  2020 ) OR  LIMIT-TO ( PUBYEAR ,  2019 )  OR  LIMIT-TO ( PUBYEAR ,  2018 )  OR  LIMIT-TO ( PUBYEAR ,  2017 )  OR  LIMIT-TO ( PUBYEAR ,  2016 )  OR  LIMIT-TO ( PUBYEAR ,  2015 )  OR  LIMIT-TO ( PUBYEAR ,  2014 )  OR  LIMIT-TO ( PUBYEAR ,  2013 )  OR  LIMIT-TO ( PUBYEAR ,  2012 )  OR  LIMIT-TO ( PUBYEAR ,  2011 )  OR  LIMIT-TO ( PUBYEAR ,  2010 ) | 865 |
