# Supplementary material for: Barriers to Breast, Cervical, and Colorectal Cancer Screenings Faced by Refugees Resettled in the United States: A Rapid Review
Source: J Immigr Minor Health. 2025 May 28;27(4):609–22. doi: 10.1007/s10903-025-01690-1 (PMC12255539; doi:10.1007/s10903-025-01690-1)
Supplement: Supplementary file 2 — Supplementary Material 2 [file 10903_2025_1690_MOESM2_ESM.docx]

**Supplemental Table 2.** Demographics of refugee populations included in studies

| **Author (year)** | **Sample size** | **Sex (M/F)** | **Age (years)** | **Employment status** | **Education level** | **Years in the U.S.** | **Years in refugee camp or second country before U.S. resettlement** | **English fluency (self-reported)** | **Marital status** | **Religion** | **Health insurance status** |
| --- | --- | --- | --- | --- | --- | --- | --- | --- | --- | --- | --- |
| **Afghani refugees** | | | | | | | | | | | |
| Shirazi et al. (2012) | 53 | **F** | **Mean**: 46  **Range**: 40-87 years | **Employed:** 10%  **Not employed:** 90% | **> 12 years:** 12%  **< 12 years:** 48%  **No formal education:** 40% | **Mean:** 16 years **Range:** 1-28 years | Not reported | **Very limited:** 40%  **No English at all:** 30% | **Married:** 65%  **Widowed:** 35% | **Muslim:** 100% | **Insured:** 77%  **Not insured:** 23% |
| **Arabic Speaking Refugees** | | | | | | | | | | | |
| Al Abdul Kader at al. (2023) | 20 | **F** | **Mean (SD):** 36.9 (11.1)  **Range:** 21-63 | **Homemaker:** 12 (60%)  **Employed:** 3 (15%)  **Self-employed:** 0  **Student:** 1 (5%)  **Unable to work:** 4 (20%) | **High school or less:** 18 (90%)  **Some college**: 1 (5%)  **College degree or higher:** 1 (5%) | Not reported | Not reported | Not reported | **Single, never married:** 2 (10%)  **Married:** 14 (70%)  **Divorced:** 0  **Widowed:** 4 (20%) | Not reported | Not reported |
| **Asian refugees & non-refugee immigrants: Chinese (13%), Vietnamese (11.6%), Karen (5.8%), Japanese (15.9%), Laos (12.6%), Nepali (17.4%), Filipino (23.7%)** | | | | | | | | | | | |
| Wu et al. (2017) | 217 | **M:** 85 (40.3%)  **F:** 126 (59.7%) | **Mean (SD):** 54 (16) | **Full time:** 99 (47.6%)  **Part time:** 26 (12.5%)  **Not employed:** 35 (16.8%)  **Retired:** 48 (23.1%) | **Less than high school:** 34 (16.2%)  **High school graduate or GED:** 35 (16.7%)  **Technical/vocational training:** 10 (4.8%)  **Some college:** 18 (8.6%)  **College graduate:** 73 (34.8%)  **Graduate school:** 40 (19.0%) | **< 1 year:** 18 (9.1%)  **1-3 years:** 28 (14.1%) **4-6 years:** 32 (16.2%) **7-9 years:** 15 (7.6%) **> 10 years:** 101 (51.0%) **Prefer not to answer:** 4 (2.0%) | Not reported | **Fluent:** 105 (49.5%)  **Short conversation:** 42 (19.8%)  **Basic words:** 33 (15.6%)  **I do not speak or understand English***:* 32 (15.1%) | **Married:** 150 (72.5%)  **Never married:** 32 (15.0%) **Widowed:** 20 (9.7%) **Divorced:** 4 (1.9%) **Separated:** 2 (1.0%) | Not reported | **Insured:** 178 (84.4%)  **Not insured:** 31 (14.7%)  **Don’t know:** 2 (0.9%) |
| **Bhutanese refugees** | | | | | | | | | | | |
| Haworth et al. (2014) | 42 | **F** | For N=37 **19-24:** 1 (2.7%) **25-34:** 5 (13.5%)  **35-44:** 12 (32.4%)  **45-54:** 13 (35.1%) **55-60:** 4 (10.8%) **60*+*:** 1 (2.7%)  **Prefer not to answer:** 1 (2.7%) | Not reported | For N=42 **Primary:** 23 (54.8%) **Secondary:** 8 (19.1%) **University/college:** 6 (14.3%) **Prefer not to answer:** 5 (11.9%) | For N=42 **<1 year:** 20 (47.6%) **1-2 years:** 11 (26.2%) **2-3 years:** 3 (7.1%) **3-4 years***:* 4 (9.5%)  **4+ years:** 3 (7.1%)  **Prefer not to answer:** 1 (2.4%) | Not reported | Not reported | For N=37 **Divorce/widow/separated:** 6 (16.2%) **Single (never been married):** 4 (10.8%)  **Married:** 25 (67.6%) **Prefer not to answer:** 2 (5.4%) | For N=37 **Hindu:** 6 (16.2%)  **Buddhist:** 9 (24.3%) **Shaman:** 2 (5.4%) **Christianity:** 14 (37.8%)  **Other/none:** 3 (8.1%)  **Prefer not to answer:** 3 (8.1%) | Not reported |
| Kue et al. (2017) | 97 | **F** | **18-24:** 21 (21.6%) **25-44:** 54 (55.7%) **45+:** 22 (33.7%) | **Employed:** 60 (61.9%)  **Not Employed:** 35 (36.1%) | Not reported | Not reported | **<10 years:** 2 (2.1%)  **10-19 years:** 45 (46.4%)  **20+ years:** 49 (50.5%) | **Not at all:** 13 (13.4%)  **Not too well:** 30 (30.9%)  **Read or speak or write well:** 53 (54.6%) | **Married:** 78 (80.4%)  **Divorced/separated/widowed:** 6 (6.2%)  **Never married:** 12 (12.4%) | **Hindu:** 74 (76.3%)  **Other:** 20 (20.6%) | **Employer:** 14 (12.4%)  **Medicare/Medicaid:** 72 (74.2%)  **None:** 8 (8.2%) |
| Lor et al. (2018) | 27 | **F** | **Mean:** 41  **20-29:** 11 (41%)  **30-39:** 0 (0%)  **40-49:** 7 (26%)  **50+:** 9 (33%) | Not reported | ≤ **1 year:** 13 (49%)  **2-6 years:** 5 (19%)  **7-11 years:** 3 (11%)  **≥ 12 years:** 6 (22%)  **No answer:** 0 (0%) | **0-4 years:** 13 (48%)  **5-9 years:** 14 (52%) | Not reported | **Fluent or well:** 4 (15%)  **Some:** 11 (41%)  **Poor or not at all:** 12 (44%) | **Married:** 22 (82%)  **Divorced/separated/widowed:** 5 (18%) | Not reported | Not reported |
| **Bosnian refugees** | | | | | | | | | | | |
| Saadi et al. (2015) | 20 | **F** | **Mean:** 54.45  **Range:** 41-75  **<30:** 0  **30-39:** 0  **40-49:** 9  **50-59:** 5  **>59:** 6 | Not reported | **Range:** middle school to college | **Mean:** 10.1  **Range:** 5-15 years **<1 year:** 0  **1-5 years:** 1  **6-10 years:** 5  **≥ 11 years:** 14 | Not reported | **None/low:** 10  **Intermediate or higher:** 10 | **Married:** 12  **Single/divorced/widowed:** 8 | **Muslim:** 19  **Christian:** 1 | Not reported |
| **Burmese refugees** | | | | | | | | | | | |
| Lor et al. (2018) | 31 | **F** | **Mean:** 34  **20-29:** 12 (39%)  **30-39:** 10 (32%)  **40-49:** 4 (13%)  **50+:** 5 (16%) | Not reported | ≤ **1 year:** 8 (26%)  **2-6 years:** 4 (13%)  **7-11 years:** 14 (45%)  **≥ 12 years:** 4 (13%)  **No answer:** 1 (3%) | **0-4 years:** 20 (65%)  **5-9 years:** 11 (35%) | Not reported | **Fluent or well:** 0 (0%)  **Some:** 9 (29%)  **Poor/not at all:** 22 (71%) | **Married:** 23 (74%)  **Divorced/separated/widowed:** 8 (26%) | Not reported | Not reported |
| Schuster et al. (2019) | 15 | **F:** 87%  **M***:*13% | **Mean:** 36.6  **SD:** 10.0  **Range***:* 22-59 | **Childcare (own):** 45%  **Cleaning/housekeeping:** 0%  **Disability/illness:** 18%  **Other:** 27%  **Unemployed:** 9% | **Never attended:** 8%  **8^th^ grade or less:** 69%  **High school:** 23% | **Mean:** 2.3 years **SD:** 1.1 years | **Mean:** 13.2 years  **SD:** 6.6 years  **Range:** 7-26 years | **Very limited:** 54%  **Limited:** 38% **Good:** 8%  **Fluent:** 0% | **Married/living together:** 100% | **Muslim:** 0%  **Christian:** 100% | Not reported |
| **Cambodian and Laos Refugees** | | | | | | | | | | | |
| Kue at al. (2021) | 22 | **F** | Mothers **Mean (SD):**56.6 (5.3)  Daughters  **Mean (SD):** 31.7 (8.5) | Mothers  **Full time:** 5 (45.5%)  **Part time:** 6 (54.5%)  Daughters  **Full time:** 9 (81.2%)  **Part time:** 2 (18.2%) | Mothers  **None:** 1 (9.1%)  **K-8th grade:** 6 (54.5%)  **12th grade graduate:** 1 (9.1%)  **Some college:** 5 (45.5%)  Daughters:  **Some college:** 5 (45.5%)  **College graduate:** 6 (54.5%) | Not reported | Not reported | Mothers*:  **Mean (SD):** 2.41 (0.85)  Daughters*:  **Mean (SD):** 3.56 (0.72)  *****Language acculturation Likert scale | Mothers  **Single, never married:** 10 (90.0%)  **Married:** 1 (9.1%)  Daughters  **Single, never married**: 3 (27.3%)  **Married:** 6 (54.5%)  **Widowed/Divorced/Separated:** 2 (18.2%) | Mothers  **Buddhist:** 11 (100%)  Daughters  **Buddhist:** 9 (81.8%)  **None:** 2 (18.2%) | Mothers  **None:** 7 (63.6%)  **Work/spouse's work**: 4 (36.6%)  Daughters  **None:** 1 (10%)  **Work/spouse's work:** 7 (70%)  **Medicare/Medicaid:** 2 (20%) |
| **Iraqi refugees** | | | | | | | | | | | |
| Saadi et al. (2012 & 2015) | 20 | **F** | **Mean:** 41.25  **Range:** 23-55  **<30:** 3  **30-39:** 4  **40-49:** 9  **50-59:** 4  **>59:** 0 | Not reported | **None:** 7  **Elementary school:** 0  **High school:** 11  **College:** 2 | **Mean:** <1  **Range:** 1mo-3 years **<1 year:** 11  **1-5 years:** 9  **6-10 years:** 0  **≥ 11 years***:* 0 | Not reported | **None/low:** 18  **Intermediate or higher***:* 2 | **Married:** 15  **Single/divorced/widowed:** 5 | **Muslim:** 19  **Christian:** 1 | Not reported |
| **Middle Eastern and Sub-Saharan African Refugees: Sudanese (11%), Somalian (15%), Kenyan (4%), Ethiopian (6%), Eritrean (6%), Congolese (15%), Ugandan (4%), Syrian (30%), Iraqi (4%), Egyptian (4%), Moroccan (2%)** | | | | | | | | | | | |
| Ghebrendrias et al. (2021) | 53 | **F** | **Range:** 20-50 | Not reported | Not reported | Not reported | Not reported | Not reported | Not reported | Not reported | Not reported |
| **Somali refugees** | | | | | | | | | | | |
| Saadi et al. (2015) | 17 | **F** | **Mean:** 39.9  **Range***:* 27-58  **<30:** 3  **30-39:** 3  **40-49:** 7  **50-59:** 4  **>59:** 0 | Not reported | **None:** 11  **Elementary school:** 1  **High school:** 4  **College:** 1 | **Mean:** 6.8  **Range:** 2-16 years **<1 year:** 0  **1-5 years:** 5  **6-10 years:** 9  **≥ 11 years:** 2 | Not reported | **None/low:** 15  **Intermediate or higher:** 2 | **Married:** 11  **Single/divorced/widowed:** 6 | **Muslim:** 17  **Christian:** 0 | Not reported |
| Schuster et al. (2019) | 15 | **F:** 87%  **M**: 13% | **Mean:** 32.5  **SD***:* 9.1  **Range:** 22-51 | **Childcare (own):** 13%  **Cleaning/housekeeping:** 27%  **Disability/illness:** 0%  **Other:** 33%  **Unemployed:** 27% | **Never attended:** 53%  **8^th^ grade or less:** 27%  **High school:** 20% | **Mean:** 6.2 years **SD:** 0.7 years | **Mean:** 12.1 years  **SD:** 2.4 years  **Range:** 8-16 years | **Very limited:** 33%  **Limited:** 40% **Good:** 27%  **Fluent:** 0% | **Married/living together:** 73%  **Separated/Divorced:** 13%  **Widowed:** 7%  **Single:** 7% | **Muslim:** 100%  **Christian:** 0% | Not reported |
| Allen et al. (2019) | 31 | **F** | **Mean:** 36  **20-29**: 9 (29%)  **30-39:** 13 (42%)  **40-49:** 4 (13%)  **50-59:** 3 (10%)  **60-69:** 2 (6%) | Not reported | Not reported | **Mean: 13 years**  **<10:** 6 (19 %)  **>10** 23 (74%) | Not reported | Not reported | **Never married:** 0  **Married or partnered:** 22 (71%)  **Separated or divorced:** 6 (19%)  **Widowed**: 2 (6%)  **Other:** 1 (3%) | Not reported | **Yes:** 31 (100%)  **No:** 0 |
